# Data-Driven Anomaly Detection Approach for Time-Series Streaming Data

**DOI:** 10.3390/s20195646

**Published:** 2020-10-02

**Authors:** Minghu Zhang, Jianwen Guo, Xin Li, Rui Jin

**Affiliations:** 1 Key Laboratory of Remote Sensing of Gansu Province, Northwest Institute of Eco-Environment and Resources, Chinese Academy of Sciences, Lanzhou 730000, China; zhangmh@lzb.ac.cn (M.Z.); jinrui@lzb.ac.cn (R.J.); 2 University of Chinese Academy of Sciences, Beijing 100049, China; xinli@itpcas.ac.cn; 3 Jiangsu Center for Collaborative Innovation in Geographical Information Resource Development and Application, Nanjing 210023, China; 4 National Tibetan Plateau Data Center, Institute of Tibetan Plateau Research, Chinese Academy of Sciences, Beijing 100101, China; 5 CAS Center for Excellence in Tibetan Plateau Earth Sciences, Beijing 100101, China

**Keywords:** wireless sensor network, environmental monitoring, anomaly detection, fault diagnosis, data mining

## Abstract

Recently, wireless sensor networks (WSNs) have been extensively deployed to monitor environments. Sensor nodes are susceptible to fault generation due to hardware and software failures in harsh environments. Anomaly detection for the time-series streaming data of sensor nodes is a challenging but critical fault diagnosis task, particularly in large-scale WSNs. The data-driven approach is becoming essential for the goal of improving the reliability and stability of WSNs. We propose a data-driven anomaly detection approach in this paper, named median filter (MF)-stacked long short-term memory-exponentially weighted moving average (LSTM-EWMA), for time-series status data, including the operating voltage and panel temperature recorded by a sensor node deployed in the field. These status data can be used to diagnose device anomalies. First, a median filter (MF) is introduced as a preprocessor to preprocess obvious anomalies in input data. Then, stacked long short-term memory (LSTM) is employed for prediction. Finally, the exponentially weighted moving average (EWMA) control chart is employed as a detector for recognizing anomalies. We evaluate the proposed approach for the panel temperature and operating voltage of time-series streaming data recorded by wireless node devices deployed in harsh field conditions for environmental monitoring. Extensive experiments were conducted on real time-series status data. The results demonstrate that compared to other approaches, the MF-stacked LSTM-EWMA approach can significantly improve the detection rate (DR) and false rate (FR). The average DR and FR values with the proposed approach are 95.46% and 4.42%, respectively. MF-stacked LSTM-EWMA anomaly detection also achieves a better F_2_ score than that achieved by other methods. The proposed approach provides valuable insights for anomaly detection in WSNs by detecting anomalies in the time-series status data recorded by wireless sensor nodes.

## 1. Introduction

The in situ deployment of large numbers of wireless sensor nodes in areas of interest plays an increasingly important role in the sensing environment owing to recent developments and trends in wireless sensor networks (WSNs). The WSN enables easy deployment of networked environment monitoring devices, which are able to collect various data and transfer the collected data to datacenters through the Internet in real time [1,2,3]. In IoT-enabled applications, WSNs are the most important component of the global earth observation system of systems (GEOSSs), since fundamental information on both the surrounding environment and the system operation status is recorded by networked sensor nodes [4,5]. In general, a wireless node consisting of a datalogger that connects with various sensors provides the service of environmental monitoring. The environment monitoring devices are widely deployed in open and unprotected extreme environments, and thus these deployed devices easily generate device anomalies [6,7]. Through real-time anomaly detection, we can find an anomaly in an instrument, repair it in time, and then guarantee data continuity. Thus, a solution that can improve the monitoring services of WSNs is needed.

The continuous measurements of both the surrounding environment and the system operation status that are recorded by deployed sensor nodes can be seen as time-series streaming data. The measurements generally include temperature, precipitation, humidity, snow depth, and device status, which are continuously transmitted to a datacenter or saved to a local device in real time. The status data of the sensor node, such as the operating voltage and panel temperature, are important indicators of whether the devices and systems are working properly, thus providing valuable information that can be exploited for finding abnormal devices and repairing devices in time. For example, the large amount of historical status data recoded by such deployed sensor nodes can be analyzed to perform fault diagnosis in the system by detecting anomalies that deviate from historical patterns. The appearance of data anomalies in the status data, such as the operating voltage and panel temperature, generally indicates that the operational state of the node device is drifting away from equilibrium for a short time period and that the sensor node may be abnormal, which indicates an imminent impact on the WSN. Therefore, analysis of status data analysis is important to ensuring the reliability of environmental monitoring [8,9,10].

Anomaly analysis of the operating voltage and panel temperature of the sensor node is one of the main challenges in large-scale WSNs. Many faults of WSNs have occurred due to voltage collapse and instability. Sensor nodes are required in order to maintain a stable operating voltage for WSN stability and reliability. The panel temperature is also an important indicator of the stable operation of node devices. An important problem in WSNs is the occurrence of failure to monitor device anomalies in a timely manner, ultimately compromising the data continuity recorded by the wireless sensor node.

Therefore, as the penetration of WSNs increases, it is crucial to detect the sensor node status and activate emergency control measures in a timely manner. Traditionally, anomaly detection uses artificial means assisted by visual data tools [11]. Researchers have proposed statistical and machine learning approaches in recent years [12,13,14,15], such as one-class support Tucker machines, and neural networks and classification. Although some anomaly detection mechanisms have been proposed, it is very difficult for them to satisfy the anomaly detection requirements with the status data of the sensor node. Therefore, to ensure the lifetime of WSNs, a novel anomaly detection approach is required that can effectively detect the deviation of status data in a WSN. The goal of this work is to detect anomalies in time-series status data recorded by sensor nodes in a WSN. These anomalies can be considered as possible faults in the wireless sensor node, which should be detected or reported by an automated detection system.

To address the above question, we introduce a data-driven anomaly detection approach to detecting anomalies in WSNs, which is to use prediction-based anomaly detectors as a replacement for traditional manual detection. In other words, we employ the median filter (MF) algorithm as a preprocessor to preprocess obvious anomalies and the stacked long short-term memory (LSTM) as a predictor. The exponentially weighted moving average (EWMA) control chart algorithm is employed as a detector to detect anomalies. We propose the model for fault diagnosis of the deployed environment monitoring device through the detection of anomalies in the operating voltage and panel temperature. Additionally, the paper aims to improve the capability of early anomaly detection in WSNs. With this aim, we present our main contributions as follows:

- We integrate different approaches, including MF, stacked LSTM, and EWMA control chart, to improve the performance of detecting possible anomalies and the occurrence of future faults in WSNs.
- We perform a comparison of the predictor and detector in a data-driven manner using three error metrics. The stacked LSTM predictor is compared to LSTM and nonlinear autoregression with the external input (NARX) neural network predictor. The MF-stacked LSTM-EWMA is also compared with the MF-LSTM-EWMA and MF-NARX-EWMA approaches.

The results of the evaluations show that the proposed approach has high efficacy. The proposed approach is the only one that investigates a data-driven anomaly detection approach for real-world status data, including the panel temperature and operating voltage recorded by a deployed sensor node in a WSN. The proposed approach can be used in similar scenarios.

This paper analyzes the related work in Section 2. The framework, detailed implementation and applied methods are presented in Section 3. Section 4 presents the dataset description and experimental results. Discussions are presented in Section 5. Finally, the conclusions are given in Section 6.

## 2. Related Work

Recently, for time-series streaming data, previous research has attracted widespread attention in terms of anomaly detection. Many theoretical and experimental works in previous surveys verify that probabilistic techniques and machine learning are highly relevant to streaming data.

Buzzi-Ferraris et al. [16] introduced a Dempster–Shafer theory-based anomaly detection approach to detect anomalies in network streaming data. Samaan et al. [17] proposed a statistical analysis-based anomaly detection method for time series in network streaming data. Ibidunmoye et al. [18] introduced a statistical hypothesis testing-based adaptive detection approach for diagnosing anomalies in performance metric streams. Fauconnier et al. [19] studied autoregressive integrated moving average (ARIMA) models for anomaly detection. These probabilistic-based approaches enable calculation simplification, and accurate detection results are obtained if the data follow the hypothesis distribution. However, the disadvantage is that the distribution of data is unknown in real datasets.

Moreover, previous works have promoted the detection approach to detect anomalies using machine learning. Akouemo et al. [20] proposed a nonlinear autoregression with external input (NARX)-based approach to approve the data quality and used the artificial neural network (ANN) to compare the performance for improved data quality with NARX. Chandola et al. [21] reported some anomaly detection approaches, including a machine learning-based approach, semi-supervised hybrid approach and window-based approach, to detect anomalies in streaming datasets. The anomalies for each observation in a test time series are equal to the differences between the predicted and actual observations. Although intelligent algorithms have been studied and have been widely used in specific application environments, use of these approaches to perform anomaly detection in status data of sensor nodes is difficult because these status data have stationary and nonstationary characteristics.

Therefore, to move toward to time-series status data in WSNs, we propose a data-driven anomaly detection approach called MF-stacked LSTM-EWMA in this paper to solve the problem of anomaly detection.

## 3. Framework and Methods

**Figure 1:**
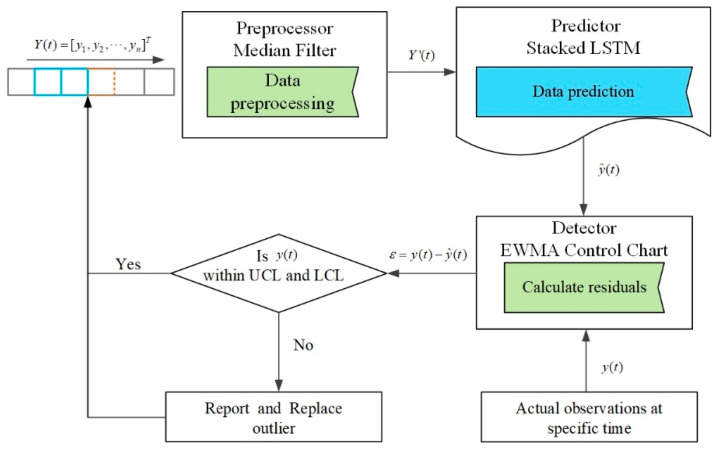
Framework of the proposed approach.

This section presents the data-driven approach called MF-stacked LSTM-EWMA for anomaly detection, which aims to increase the efficiency of anomaly detection efforts in WSNs. The MF preprocesses obvious anomalies in the streaming data. Stacked LSTM was used as a predictor to predict the expected value. The predictor first makes a prediction $\hat{y}(t)$ using a sliding window built from raw data Y(t). Thus, the raw value y(t) is based on the defined time window for obtaining the predicted value $\hat{y}(t)$. To detect the anomalies, we calculate the conditional residuals, $\varepsilon (t)=(y(t)-\hat{y}(t))$, which show the differences between the prediction and raw data at a specific time. The EWMA algorithm is employed to detect and report anomalies in the residuals, and the identified anomalies are replaced using the predicted expected value. The framework of the proposed MF-stacked LSTM-EWMA approach is presented in Figure 1.

In brief, MF-stacked LSTM-EWMA includes five steps: In step 1, a preprocessing algorithm takes the raw historical data as the input to preprocess obvious anomalies. In step 2, the preprocessed data are input to the predictor to obtain the predicted values. In step 3, finally, the conditional residuals ε(t) are calculated. Note that the specific time is defined based on the defined sliding window. We classify the value at a specific time based on the detector. In step 4, if the value is detected as an anomaly, it is reported to the maintenance personnel of the devices as an early warning to ensure the normal operation of the WSN over a long lifetime. In step 5, steps 1–4 are repeated.

### 3.1. MF-Based Preprocessor

In the first step of the data-driven approach for anomaly detection, the MF algorithm is used to preprocess anomalies in the data stream [21,22,23]. First, if input data with anomalies are imported into a prediction model, inaccurate predictions will be generated. Thus, anomalous input data must be detected before they are imported into the prediction model so that the detector can provide a better processing probability. Second, the time series is presented as $Y(t)=[y_{1},y_{2},\ldots ,y_{n}]$, where *n* represents the total number of the series. Y(t) is input into MF for preprocessing the obvious anomalies. This method assumes that the moving window is defined as m. In the proposed approach, we set m to 5 [21].

### 3.2. Stacked LSTM-Based Predictor

Next, the stacked LSTM is employed as a predictor for forecasting, which is the raw value based on the defined time window [24,25,26]. The LSTM model was proposed in 1997, and it shows certain advantages in dealing with long-term time-series data [27,28,29]. Stacked LSTM, based on LSTM, improves the training efficiency and obtains higher accuracy by adding depth to the network. The stacked LSTM model consists of multiple LSTM layers. Moreover, like the LSTM model, the stacked LSTM model obtained the prediction value $\hat{y}(t)$ based on the value of y(t). Benefiting from the key advantage of the LSTM, we use the stacked LSTM model to deal with anomalous occurrences in time-series data.

**Figure 2:**
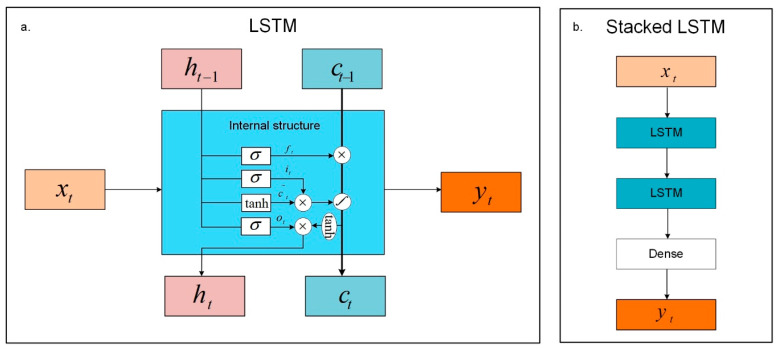
The structure of long short-term memory (LSTM) and stacked LSTM. (**a**). The structure of LSTM, including the internal structure; (**b**) the stacked LSTM structure with two LSTM layers.

The structure of LSTM and stacked LSTM are shown in Figure 2, and the structure of LSTM, including the internal structure is shown in Figure 2a. The calculation process includes 6 steps.

1. Calculate ${\tilde{c}}_{t}$.
  (1) $${\tilde{c}}_{t}=\tanh(W_{c}\cdot [h_{t-1},x_{t}]+b_{c})$$
  where ${\tilde{c}}_{t}$ represents the cell status, $W_{c}$ represents the weight matrix, $h_{t-1}$ represents the output, $x_{t}$ presents the input at time *t* and $b_{c}$ is the bias.
2. Then, input gate $i_{t}$ is calculated.
  (2) $$i_{t}=\sigma (W_{i}\cdot [h_{t-1},x_{t}]+b_{i})$$
  where σ represents the sigmoid function, $W_{i}$ is the weight matrix, and $b_{i}$ is the bias.
3. Next, forget gate $f_{t}$ is calculated.
  (3) $$f_{t}=\sigma (W_{f}\cdot [h_{t-1},x_{t}]+b_{f})$$
  where $W_{f}$ represents the weight matrix and $b_{f}$ represents the bias.
4. Calculate $c_{t}$.
  (4) $$c_{t}=f_{t}*c_{t-1}+i_{t}*c_{t}$$
  where ‘‘*’’ represents the dot product, $c_{t}$ represents the current cell, and $c_{t-1}$ represents the last cell.
5. The output gate $o_{t}$ is calculated.
  (5) $$o_{t}=\sigma (W_{o}\cdot [h_{t-1},x_{t}]+b_{o})$$
  where $W_{o}$ represents the weight matrix, and $b_{o}$ represents the bias.
6. Finally, the output $h_{t}$ is calculated.
  (6) $$h_{t}=o_{t}*\tanh(c_{t})$$

Benefiting from the merits of LSTM, stacked LSTM is introduced as a predictor for improving the prediction accuracy of the time-series data. It is important to note that the stacked LSTM model includes two LSTM layers, and the flow chart of the stacked LSTM model is presented in Figure 2b.

### 3.3. EWMA Control Chart-Based Detector

Statistical techniques such as the EWMA control chart show robustness in detecting anomalies in time-series streaming data [18,30]. Unlike other control charts such as Shewhart’s, the EWMA control chart is a process control mechanism for monitoring variability [28]. Thus, we introduce the EWMA control chart as a detector for diagnosing anomalies in status data. The control chart consists of two upper and lower control limits, namely, the UCL and LCL. The centerline (CL) is calculated by the mean of the conditional residuals, $\varepsilon (t)=(y(t)-\hat{y}(t))$. $\varepsilon_{t}=\lambda \varepsilon_{t}+(1-\lambda )\varepsilon_{t-1}$ represents EWMA control chart, where 0<λ<1. The function of λ is to exponentially smooth the prediction residuals. $(UCL_{t},LCL_{t})=\mu \pm L\sigma_{t}\sqrt{\frac{\lambda }{2-\lambda }[1-{\left(1-\lambda \right)}^{2t}]}$ shows the CL, UCL and LCL, where L represents the regulatory factor that is used to control the sensitivity of the control chart, $\mu_{t}$ represents the arithmetic mean, and $\sigma_{t}$ is the standard deviation. We employ the EWMA control chart for detecting anomalies according to the following rule. We classify an observation value as anomalous or non-anomalous by calculating the residuals between the predicted value and the actual observation at a specific time through the EWMA control chart. We set (λ,L) based on a confidence level of 90% [23].

## 4. Evaluation and Results

### 4.1. Research Area and Dataset

#### 4.1.1. Research Area

**Figure 3:**
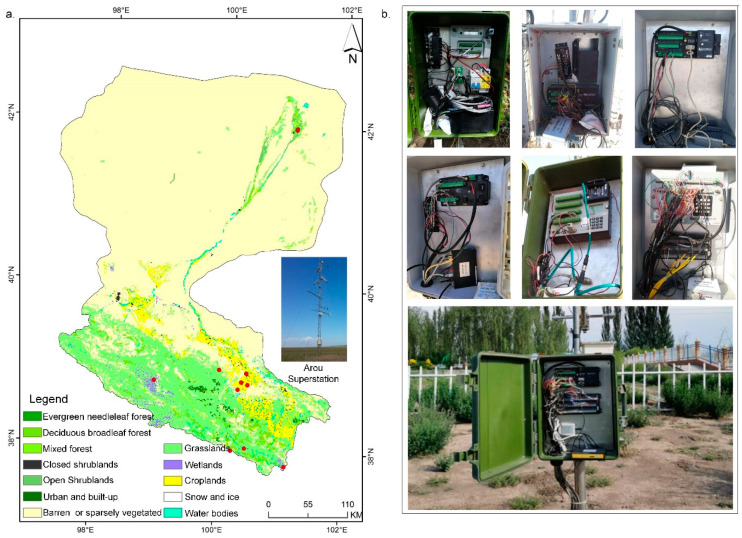
Research area and various types of dataloggers for environmental monitoring. (**a**) The location of the research area and Arou superstation. The red plots represent the locations of the 11 observation stations; (**b**) The various types of dataloggers deployed in the Heihe River basin for environmental monitoring, which consist of 4G wireless modules and sensors.

The Heihe River basin (37.7°–42.7° N, 97.1°–102.0° E) covers an area of approximately 143 × 10^3^ km^2^ [31,32,33,34]. Most of the area is cold and arid. In the past 30 years, the observation network has been constantly established and improved [35]. An eco-hydrological WSN has been established to monitor the environment and provide a data set for answering scientific questions. To date, there are 11 observation stations, and approximately 30 dataloggers have been deployed to monitor the environment in the entire research area. According to statistical data, there are more than 2147 sensors in the area for monitoring approximately 341 types of environmental variables in real time. A major challenge of in situ node device management is that the research area is vast and has a complex terrain, and many areas are difficult environments with high altitudes, so the network is very prone to generating faults. Traditional fault detection for devices is labor intensive. The location of research area and various types of dataloggers deployed in field are presented in Figure 3.

#### 4.1.2. Dataset

The proposed approach is evaluated in a data-driven manner. We conduct experiments to detect anomalies in in situ sensor nodes deployed in the research area by detecting anomalies in the time-series status data, such as the panel temperature and operating voltage that were collected from the deployed dataloggers. The Arou superstation is located at 38.0384° N, 100.4572° E, where the altitude is 3033 m. The node devices deployed at Arou station are prone to faults due to their high altitude and harsh environment. Thus, we select the status data recorded by the datalogger deployed in the Arou superstation. In the Arou superstation, many monitoring devices are deployed, such as 16 soil moisture WSN nodes, an eddy-covariance system, a weighing-type rain gauge, 2 large-aperture scintillometers, and a vegetation phenology observation system. The details of the deployed environment monitoring devices are introduced by Li et al. [31].

In this paper, we select the time-series status data recorded by the CR1000 for diagnosing faults by detecting anomalies in the status data. The selected panel temperature and operating voltage data are collected in the time range from 1 November 2019 to 10 November 2019 (*n* = 1440), and the time interval of data collection is 10 min.

**Figure 4:**
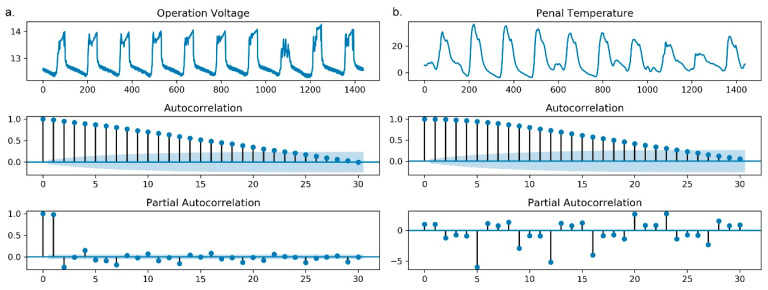
The plots of the autocorrelation function (ACF) and partial autocorrelation function (PACF) of the operating voltage and panel temperature calculated from 30 logs. (**a**) The ACF and PACF of the operating voltage; (**b**) the ACF and PACF of the panel temperature.

The results of the autocorrelation function (ACF) and the partial autocorrelation function (PACF) on the panel temperature and operating voltage calculated from 30 logs are presented in Figure 4. According to the figure, the 0th-order autocorrelation coefficient and 0th-order partial autocorrelation coefficient are constant at 1. In Figure 4a, the autocorrelation coefficient and partial autocorrelation coefficient decrease rapidly from 1 to nearly 0, and the partial autocorrelation coefficient fluctuates slightly up and down on the 0 axis with the increase in order, which largely meets the requirements of stationarity. In Figure 4b, the autocorrelation coefficient decreases rapidly from 1 to near 0; however, the partial autocorrelation coefficient fluctuates violently up and down the 0 axis with the increase in order. The findings suggest that the operating voltage shows an obvious stationary process, and the panel temperature shows a nonstationary process.

Note that some artificial anomalies are injected to evaluate the anomaly detection approaches. For evaluation of the anomaly detection approach, this is a common approach in previous studies [36]. Thus, we randomly injected some anomalies that have slightly deviations from the historical trend in the test data.

### 4.2. Evaluation Metrics

The proposed MF-stacked LSTM-EWMA approach for detecting anomalies includes three steps. Thus, we evaluate the approach in three stages. To evaluate the performance of the preprocessor and predictor, three error metrics—the mean squared error (MSE), mean absolute error (MAE), and root-mean-square error (RMSE)—are used [37,38]. Their formulas are presented in Equations (7)–(9).

(7) $$MSE=\frac{1}{n}\sum_{t=1}^{n}(y(t)-\tilde{y(t)})$$

(8) $$MAE=\frac{1}{n}\sum_{t=1}^{n}|y(t)-\tilde{y(t)}|$$

(9) $$RMSE=\sqrt{\frac{1}{n}\sum_{t=1}^{n}(y(t)-\tilde{y(t)}{)}^{2}}$$

In addition, the Taylor diagram was employed to evaluate the performance of the predictor. A Taylor diagram is a kind of diagram that can express the three indexes of the standard deviation, root-mean-square deviation (RMSD) and correlation coefficient [39]; it can display the standard deviation, the correlation coefficient with a reference value and the root-mean-square deviation on a two-dimensional graph, and it can comprehensively and clearly reflect the performance of various models. Therefore, the Taylor diagram has been widely used as an effective method for model evaluation.

Finally, we evaluate the performance of the proposed MF-stacked LSTM-EWMA using DR = TP/(TP + FN), PR = TP/(TP + FP), FR = FP/(FP + TN), and F_β_ = (β_2_ + 1) (PR × DR)/(β_2_ × PR + DR) [36,40,41], where DR is the detection rate, PR is the precision rate, and FR is the false rate. In the prediction-based detector, given a predicted value and a raw value, four different outcomes are present, including TP, FP, TN, and FN. TP indicates that the raw value is anomalous and that it is detected as anomalous; FN indicates that the raw value is anomalous and that it is detected as non-anomalous. If the raw value is non-anomalous and it is detected as non-anomalous, it is recorded as a TN; if it is detected as anomalous, it is recorded as a FP [42]. The F_β_- score is a performance index that weighs the importance between recall and precision. In this paper, we set β = 2 according to Ibidunmoye et al. [18].

### 4.3. Performance Evaluation

In the framework of proposed MF-stacked LSTM-EWMA approach, the MF algorithm takes the raw data as input to preprocess obvious anomalies in the first step. The stacked LSTM approach is used to establish a predictor in the second step. The last step identifies anomalies via the EWMA control chart.

#### 4.3.1. Evaluation of the Preprocessor

**Figure 5:**
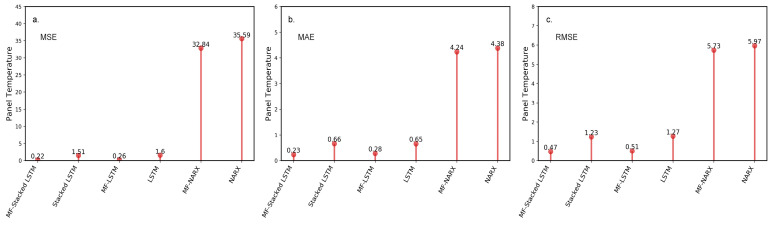
The resulting mean squared error (MSE), mean absolute error (MAE), and root-mean-square error (RMSE) with different approaches for the panel temperature. (**a**) The results for the MSE; (**b**) The results for the MAE; (**c**) The results for the RMSE.

The performance of the MF algorithm on preprocessing obvious anomalies is evaluated in this section. We calculate the error metrics between the predicted value and the raw data, including the raw (un-preprocessed) data and the data preprocessed by the MF algorithm. The un-preprocessed and preprocessed data, including the panel temperature and operating voltage (*n* = 1202), are used to train the predictor. We compare the effect of applying the MF algorithm to the predictor on the prediction accuracy. Figure 5 shows the MSE, MAE, and RMSE calculated for the un-preprocessed and preprocessed panel temperature and operating voltage for different predictors.

For the panel temperature, the evaluated results are shown in Figure 5. Figure 5a–c show that the three error metrics calculated for the preprocessed data are smaller than the metrics calculated for un-preprocessed data. For example, we comapre the MF-stacked LSTM with stacked LSTM, an approach without a preprocessing step. MF-stacked LSTM yields an observed improvement over stacked LSTM, with MSE, MAE and RMSE values of approximately 85.49%, 65.15%, and 61.79%, respectively. Similarly, for the MF-LSTM approach, compared with LSTM, there is an average of 83.75% improvement in the MSE, a 56.92% improvement in the MAE and a 59.84% improvement in the RMSE. Compared to the NARX approach, MF-NARX provides an average improvement of 7.68% in the MSE, 3.2% in the MAE and 4% in the RMSE.

**Figure 6:**
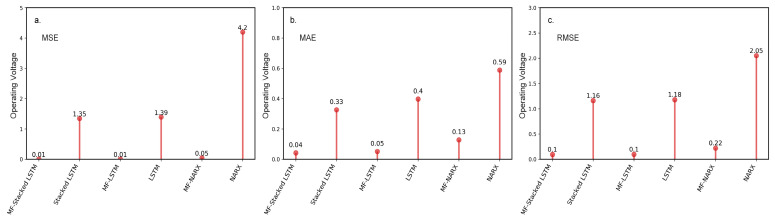
The resulting MSE, MAE, and RMSE with different approaches for the operating voltage. (**a**) The results for the MSE; (**b**) The results for the MAE; (**c**) The results for the RMSE.

Similar to the panel temperature, we evaluate the MF algorithm on operating voltage data. The results for the MSE, MAE, and RMSE are shown in Figure 6. According to Figure 6a–c, the findings derived from the predictors with the MF are compared to those of the predictors without the MF. The predictors with the MF algorithm outperform the predictors without the MF algorithm in terms of three error metrics. We conclude that the results are consistent. It is clear that the anomalies can obviously affect the performance of the predictors, and the MF can improve the prediction accuracy efficiently.

#### 4.3.2. Evaluation of the Predictors

Additionally, we evaluate the performance of MF-stacked LSTM, MF-LSTM, and MF-NARX as predictors for the time-series data stream. The resulting MSE, MAE, and RMSE for different predictors for the panel temperature and operating voltage are presented in Table 1.

For the panel temperature, we report the performance of MF-stacked LSTM compared to MF-LSTM; on average, there is a 15.38% improvement in the MSE, a 17.85% improvement in the MAE, and a 7.84% improvement in the RMSE. Likewise, we compared MF-stacked LSTM with the MF-NARX approach. MF-stacked LSTM obtains the maximum observed improvement, with MSE, MAE, and RMSE values of approximately 99.33%, 94.57%, and 91.80%, respectively. It is obvious that the accuracy of MF-stacked LSTM is quite high, suggesting that the prediction accuracy is dominated by the predictors.

Moreover, for the operating voltage, we also report the performance of MF-stacked LSTM compared with MF-LSTM and MF-NARX. The findings suggest that the employed predictor yields a higher prediction accuracy than MF-LSTM and MF-NARX. The evaluation for the operating voltage suggest that when we transform the test data, MF-stacked LSTM still obtains a high prediction accuracy. Interestingly, while MF-stacked LSTM shows good performance in predicting the time-series data stream, we also note the performance of MF-NARX for the panel temperature and operating voltage. For the operating voltage, MF-NARX shows potential in its performance. However, its use is hampered for the panel temperature, i.e., MF-NARX shows obvious sensitivity with respect to different types of data.

**Figure 7:**
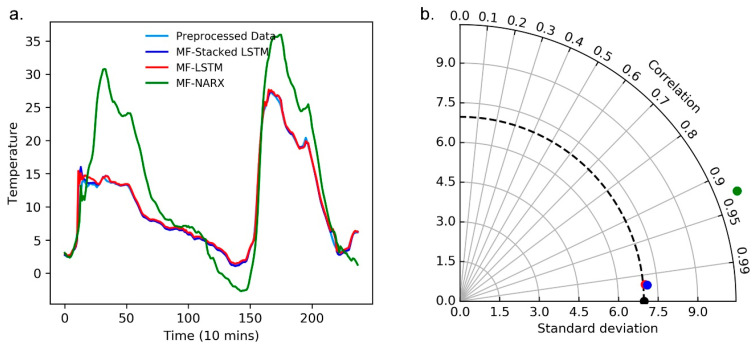
Curve diagram and Taylor diagram of the predictors with the median filter (MF) for the panel temperature. (**a**) The curve diagram of the predictors; (**b**) The Taylor diagram of the predictors.

**Figure 8:**
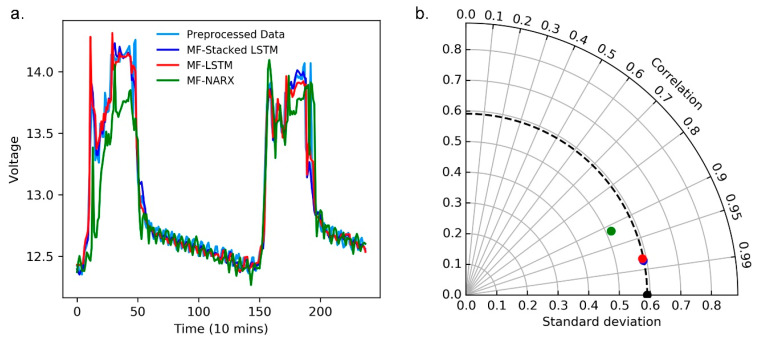
Curve diagram and Taylor diagram of the predictors with the MF for the operating voltage. (**a**) The curve diagram of the predictors; (**b**) The Taylor diagram of the predictors.

Moreover, we use a curve diagram and a Taylor diagram to show the performance of MF-stacked LSTM, MF-LSTM and NARX. In Figure 7a and Figure 8a, the horizontal axes represent the number of predicted and raw values, where the raw value was collected every ten minutes, the curve diagrams show that compared to MF-LSTM and MF-NARX, the results of MF-stacked LSTM are closest to the raw data.

Note that in Figure 7b and Figure 8b, the standard deviation (SD), RMSD and correlation coefficient show the differences between the predicted and raw values, and the predicted values are predicted by MF-stacked LSTM, MF-LSTM and MF-NARX. The radii of the light gray cycles represent their values. In Figure 7b and Figure 8b, the black point is the reference point of the raw data.

We compare the performance of different predictors, such as the stacked LSTM, LSTM and NARX. Figure 7 and Figure 8 and Table 1 present the compared results. From Figure 7 and Figure 8, taking the MF-stacked LSTM output versus those of MF-LSTM and MF-NARX as an example, the result of the MF-stacked LSTM output and that of MF-LSTM are highly similar. The MF-stacked LSTM and MF-LSTM for the panel temperature and operating voltage demonstrate that R > 0.97 and RMSDs is small. However, the NARX predictor provides poor correlation results (R < 0.95) because the panel temperature has significant nonstationary features and NARX is sensitive to the type of data, this result is as expected. Moreover, it is obvious that the accuracy of anomaly detection is affected by the predictors.

#### 4.3.3. Evaluation of the Detectors

**Figure 9:**
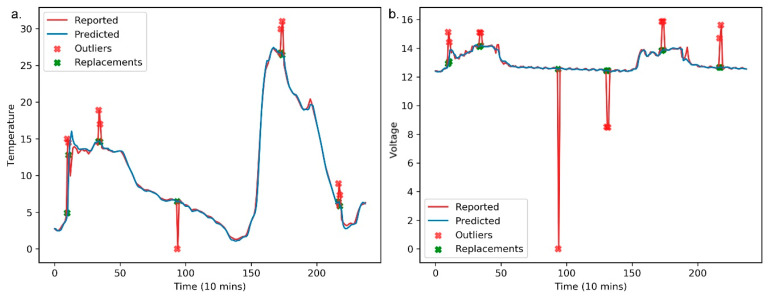
The performance of anomaly detection on panel temperature and operating voltage using the MF-stacked LSTM predictor. (**a**) The performance of anomaly detection on panel temperature; (**b**) The performance of anomaly detection on operating voltage.

Figure 9 shows the found anomalies, along with their raw and replaced values for the panel temperature and operating voltage. Similar to Figure 7 and Figure 8, the horizontal axes represent the number of predicted and raw values in Figure 9. The anomaly detection results for the panel temperature are shown in Figure 9a. The anomalies are replaced by the predicted values, and the stacked LSTM predictor appears to be particularly accurate. Additionally, we evaluate the detection performance for the operating voltage using the stacked LSTM predictor. The performance of the anomaly detector can be seen clearly in Figure 9b. It is important to note that the stacked LSTM predictor-based anomaly detector is robust due to the advantages of stacked LSTM.

Table 2 shows the comparison of the detection results for MF-stacked LSTM-EWMA, MF-LSTM-EWMA and MF-NARX-EWMA. According to the table, note that MF-stacked LSTM-EWMA and MF-LSTM-EWMA achieve consistent superiority across all dataset groups. For example, MF-stacked LSTM-EWMA has DRs for the panel temperature and operating voltage of 100% and 90.91%, respectively. The results for MF-LSTM-EWMA show that the DR remains the same as that of MF-stacked LSTM-EWMA regardless of the data type. Largely because the detector employs an LSTM-based model, it improves the prediction performance. Furthermore, MF-stacked LSTM and MF-LSTM achieve almost the same prediction accuracy in terms of the panel temperature and operating voltage. However, MF-NARX-EWMA shows a poor DR across all dataset groups. The DRs for the panel temperature and operating voltage are 0 and 100%, respectively. This is expected, since the panel temperature is nonstationary and the operating voltage is stationary. It is worth noting that compared to MF-stacked LSTM-EWMA and MF-LSTM-EWMA, although a 100% DR was obtained, it also resulted in a higher FR.

The FRs for the detectors are also shown in Table 2. According to the results, MF-stacked LSTM-EWMA provides a better FR than MF-LSTM-EWMA. For example, the FRs of MF-stacked LSTM-EWMA for the panel temperature and operating voltage are 4.57% and 4.39%, respectively, and those of MF-LSTM-EWMA for the panel temperature and operating voltage are 6.11% and 4.8%, respectively. In particular, the results for MF-NARX-EWMA show a high FR for the panel temperature, 94.14%. Similarly, the operating voltage results show that MF-NARX-EWMA results in a higher FR than MF-stacked LSTM-EWMA and MF-LSTM-EWMA. The FR of MF-NARX-EWMA is 8.77%, and those of MF-stacked LSTM-EWMA and MF-LSTM-EWMA are 4.39% and 4.8%, respectively.

The F-measure shows the advantage for comprehensive evaluation. For MF-stacked LSTM-EWMA, the F_2_ values for the panel temperature and operating voltage are 81.82% and 78.13%, respectively. The F_2_ of MF-LSTM-EWMA for the panel temperature and operating voltage are 76.27% and 76.93%, respectively. MF-NARX-EWMA obtains only 0 and 69.39% F_2_ for the panel temperature and operating voltage. According to the results, MF-stacked LSTM-EWMA achieves a performance comparable with that of MF-LSTM-EWMA and performance superior to that of MF-NARX-EWMA for the panel temperature and operating voltage. The MF-stacked LSTM-based and MF-LSTM-based detectors show better performance than the MF-NARX-based detectors in error classification. Thus, the MF-NARX-EWMA detector performs poorly at anomaly detection in the panel temperature data.

Furthermore, we compare the approaches, including MF-stacked LSTM-EWMA, MF-LSTM-EWMA, and MF-NARX-EWMA, with stacked LSTM-EWMA, LSTM-EWMA, and NARX-EWMA, which do not have a data preprocessing algorithm, e.g., an MF. The anomaly detectors without an MF show poor performance in general compared to the anomaly detectors with an MF. Their low F_2_ scores are unattractive due to their poor DRs and FRs across all test data, especially the operating voltage. For example, stacked LSTM-EWMA obtains F_2_ scores for the panel temperature and operating voltage of 35.48% and 8.57%, while compared to stacked LSTM-EWMA, MF-stacked LSTM-EWMA obtains an average of 56.63% and 89.03% improvement in F_2_ score on panel temperature and operation voltage, respectively. The use of the MF algorithm therefore significantly improves the ability of the stacked LSTM-EWMA and LSTM-EWMA detectors in correctly classifying erroneous data.

In addition, the total time of each approach, including preprocessing, training, testing, and detection time, are presented in Table 2. The table shows that compared to the detector with MF, the detector without MF uses almost the same time. The results suggest that the MF as a preprocessor requires less time to preprocess input data. However, the MF-stacked LSTM-EWMA requires more time to detect the anomaly than the MF-LSTM-EWMA. The reason for this outcome is that the stacked LSTM consists of two LSTM layers. It is worth noting that the MF-NARX-EWMA requires the least amount of time to detect the anomaly. It is noteworthy that compared to MF-stacked LSTM-EWMA and MF-LSTM-EWMA, the MF-NARX-EWMA shows an advantage in time consumption.

In summary, it is likely that the detection performance is affected by the predictors, and these factors have a multiplicative affect on the final detection results. In summary, all of the aforementioned approaches demonstrate the feasibility of anomaly detection. The results indicate that a prediction-based detector for the data stream, coupled with a preprocessing algorithm, performs well in identifying anomalies in the time-series status data recorded by node devices deployed in the research area.

## 5. Discussion

The proposed data-driven anomaly detector for detecting faults in WSNs is tested in two cases: anomaly detection for the panel temperature and for the operating voltage of wireless nodes. The detector relies on the performance of a predictor and detects anomalies by delineating the boundary between anomalous and non-anomalous data using an EWMA control chart. This type of approach is sensitive to the data quality. Thus, selecting an appropriate preprocessing method and predictor is important.

The benefit of using an MF is that the MF has high performance in processing obvious anomalies in a time-series data stream. For example, Figure 5 and Figure 6 show the benefit of using an MF. For the panel temperature, MF-stacked LSTM yields an observed improvement over stacked LSTM, with MSE, MAE, and RMSE values of approximately 85.49%, 65.15%, and 61.79%, respectively. Similarly, for the operating voltage, compared to stacked LSTM, MF-stacked LSTM provides an average improvement of 99.25% in the MSE, 87.88% in the MAE, and 91.38% in the RMSE. The results show that the MF is feasible for data preprocessing. The performance of the approaches indicates that the MF shows a significant influence on the performance of the predictor.

Note that the predictor, however, usually cannot give an accurate forecasting result for different types of data because of the stationary and nonstationary features of the test data; for example, the operating voltage shows obvious stationary features, and the panel temperature is a nonstationary process. For this reason, the predictor should be selected properly so that the prediction accuracy rate is reasonably high.

In addition to providing an accurate predictor for data prediction, the introduced anomaly detection approach is important, since the data generated by the deployed wireless nodes are streaming data. Fortunately, for prediction-based anomaly detection, the EWMA control chart yields good results for anomaly detection because the EWMA is robust for determining deviations in streams of conditional residuals, especially when it is used in prediction-based detectors.

Finally, the proposed approach can provide good performance in nonstationary and stationary time-series data, i.e., data such as the panel temperature, the pattern of which changes over time, are nonstationary, whereas data such as the operating voltage, the pattern of which do not change with time, are stationary.

## 6. Conclusions

Detecting anomalies in a time-series data stream is crucial for diagnosing faults in deployed node devices in WSNs. Thus, a data-driven anomaly detection method is introduced for detecting faults in WSNs. The proposed MF-stacked LSTM-EWMA approach integrates MF preprocessing, a stacked LSTM predictor and an EWMA control chart anomaly detector, and it achieves excellent performance in anomaly detection for time-series streaming data. To demonstrate its performance in detecting anomalies, we show the efficacy of our approaches in anomaly detection for the panel temperature and operating voltage of node devices. Compared with the MF-LSTM-EWMA and MF-NARX-EWMA approaches, the proposed MF-stacked LSTM-EWMA approach achieves better accuracy in anomaly detection and achieves the expected effect for time-series streaming data.

We currently aim to extend the proposed approach with a focus on automatically diagnosing and reporting faults on the wireless node side. By applying the proposed method to automatically predict and diagnose faults in wireless nodes deployed in difficult areas, we can increase the diagnosis ability while reducing the related costs.

## Figures and Tables

**Table 1:** The resulting MSE, MAE, and RMSE with different predictors for the panel temperature and operating voltage. MF represents the median filter, and NARX represents the nonlinear autoregression with the external input neural network.

Data	Method	with MF			without MF		
		MSE	MAE	RMSE	MSE	MAE	RMSE
Panel temperature	NARX	32.84	4.24	5.73	35.59	4.38	5.97
	LSTM	0.26	0.28	0.51	1.60	0.66	1.27
	Stacked LSTM	0.22	0.23	0.47	1.51	0.64	1.23
Operating voltage	NARX	0.05	0.13	0.22	4.20	0.59	2.05
	LSTM	0.01	0.05	0.10	1.39	0.40	1.18
	Stacked LSTM	0.01	0.04	0.10	1.35	0.33	1.16

**Table 2:** Comparison of the detection results for stacked LSTM, LSTM, and nonlinear autoregression with the external input (NARX) for the panel temperature and operating voltage.

Data	Method	with MF				without MF			
		DR (%)	FR (%)	F_2_ (%)	Time (s)	DR (%)	FR (%)	F_2_ (%)	Time (s)
Panel temperature	NARX	0	94.14	0	2.78	89.89	85.59	16.66	2.69
	LSTM	100	6.11	76.27	9.27	66.67	18.78	32.47	9.18
	Stacked LSTM	100	4.57	81.82	16.67	55.56	15.72	35.48	16.58
Operating voltage	NARX	100	8.77	69.39	2.73	0	85.59	0	2.64
	LSTM	90.91	4.8	76.93	9.3	9.09	6.18	8.61	9.21
	Stacked LSTM	90.91	4.39	78.13	16.42	9.09	5.73	8.57	16.33
